# Portions of the Address by Prof. Thomas F. Rochester, M. D., of Buffalo. Chairman of the Section on Practical Medicine before Am. Med. Association, 1879

**Published:** 1879-07

**Authors:** 


BUFFALO

### Medical and Surgical Journal.

VOL. XVIII. JULY, 1879. No. 12.

### Original Communications.

—:o:—

*ART. I.—Portions of the Address by* Prof. THOMAS F. ROCHESTER, M. D., *of Buffalo, Chairman of the Section on Practical Medicine, before the American Medical Association, at Atlanta, Ga., May 7th,* 1879.

The limitation of epidemic pestilential disease is at all times a question of intense interest to every thoughtful sanitarian, and at no time more than at present, when the vivid remembrance of last year's fearful visitation of yellow fever is but too fresh in the minds of all.

Avoiding its usual selection of localities—the cities of the Gulf of Mexico and of the southeastern seaboard—it swept the towns on the banks of the Mississippi and its tributaries with an intensity, virulence, and fatality never exceeded in any locality; neither acclimation, or previous attack, or race affording any immunity, the generally accredited exemption of the negro not holding good.

No pen can portray the suffering, the horrors and privations experienced, although many vivid pictures were drawn of the dread reality. A field for philanthropy of the highest order was opened, and it was exercised in a manner most creditable to the human heart. Brave men and devoted women sacrificed themselves by the thousand in extending relief to their fellows, and the clergy and the medical profession especially contributed most largely, by their labors and exposure, to the number of self-immolated victims. The heart of the whole nation was stirred: and when the smitten brother appealed for help, money poured in like water from a flowing stream. No place was too remote, too poor, or too small to proffer its aid; and, as the cry extended across the sea, succor was sent from London, Liverpool, Paris, and other European cities, including Stamboul—even the distressed Ottoman gladly parting with some portion of his scanty means for the cause of common humanity.

Of what avail was all this treasure? It buried the dead, and fed and clothed the destitute, and paid some of the necessary incidental expenses caused by the plague. It could do little else, for the disease held its dire sway, and was but slightly influenced by medication. In some localities and under some practitioners therapeutic measures apparently were of avail to a slight extent, but it is not disputed, as a rule, that the malady was uninfluenced by what we call medication. The all-important question then comes up, Is it possible to ward off the invasion of this pestilence, or to confine it within limits where it has acquired a foothold? To answer this necessitates a brief ætiological investigation. What is its precise and definite origin, like that of many, or more strictly most, disorders is unknown, but where it is born is known.

As surely as cholera is a product of the jungles of India and Burmah, as certainly is yellow-fever of West Indian origin. That it is an exotic as relates to the United States, is the opinion of the last National Commission; and that it never originates *de novo,* except in its primal birth-place, whatever elsewhere may be the excess of heat moisture, filth, and vegetable and animal decomposition, is almost demonstrated, perhaps established. As to communicability, it is certainly conveyed from individual to individual, not precisely by what we understand to be direct contagion, but through various media, especially by bed and body clothing, by articles of furniture, by apartments, cars and steam and sailing vessels, by baggage and by cargoes; and these propagators, deriving from the sick the pestilential material (intentionally not called germ), hold it with wonderful tenacity, and convey it to mankind with intense effect. Of this property there are numberless illustrations; one is selected to establish the truth of the above assertion. In September, 1856, an infected ship from Cuba was detained at the quarantine anchorage off Staten Island, N. Y. Several passengers had died, and some were ill on board. The garments and bedding were thrown overboard. Bay-Ridge, a delightful suburban neighbor of Brooklyn, the seat of choice country residences, lies directly across the bay, distant about one mile from the anchorage mentioned. The wind and tide deposited a number of the garments that had been thrown away, on the beach which terminated the lawn of Col. Charles Prince, an old and respected resident. In taking his usual morning walk he discovered the clothing and examined it with his cane, not otherwise handling it. He had no suspicion that it came from Quarantine, and never saw it again. In four days he was taken ill, and died in a week from yellow-fever. The man who nursed him, and who had previously had yellow-fever, took away with him the suit of clothes the Colonel was wearing, contracted therefrom the disease, and died. The son and daughter—adults—the Colonel's only children, were also attacked; the son died, and this was the commencement of an epidemic which destroyed many lives in a limited area, but which was stopped by enforced isolation and by destruction of bedding and garments. The clothing which produced all this evil had been saturated with salt-water, and had been tossed by the waves for more than twenty-four hours before it made its fatal landing.

Dr. Alfred Stillé, in a recent lecture to the graduating class of the University of Pennsylvania on this topic says: "As a single illustration of the mode in which it spread, I may cite the case of Grenada, Miss., a town of 2,500 inhabitants, of whom 1,040 were attacked with the fever, and 326, or more than 30 per cent., died. The fever first broke out in a family of which the mother had been to the railroad depot to see her daughter off to a neighboring town. The train was from New Orleans, where the fever was then raging, and the mother, it is thought, occupied a seat in the railroad car alongside her daughter for about twenty minutes, while the New

Orleans passengers were taking breakfast." It is also stated that the disease has a nearly fixed ratio of progress, apart from its infectious properties; moving at the rate of about forty feet per diem wherever it may be located.

If this be true, it is an important point to be considered, in connection with the established fact that it is arrested by a freezing temperature, indeed a continuation of a less degree of cold than 32°, for in Cuba, where there is no frost, it does not prevail in the cooler season of the year. Medicine will not cure, nor will cleanliness or antiseptics prevent, the progress of this disease.

Can anything be done that the horrors of the last and of other epidemics may be escaped in future? If there is any hope of such a consummation, does it not lie in a strictly enforced quarantine? That it need not be distressingly burdensome to individuals or greatly prejudicial to commerce is abundantly shown by the fact that it has been maintained at the port of New York for the past seven years by that most intelligent and efficient health officer, Dr. S. Oakley Vanderpoel. During this period many cases of yellow-fever have been received and treated at the quarantine hospital, and ships have been held and thoroughly purified and disinfected, both as relates to vessel and cargo, and have then been discharged from custody and allowed to proceed to the populous city, even in the hottest season of the year, without a single known instance of propagation of the disease.

The very mention of the term "quarantine" raises at once a feeling almost of anger in the minds of many. It is called barbarous, ineffectual, oppressive, a relic of ignorance and superstition, and even by some zealots an opposition to the dispensations of Providence. But hue and cry are generally irrational, and until something as effectual, or more so, is suggested a deaf ear must be turned to such protestations. Cholera has been held at bay by quarantine. In 1851 it prevailed in Southern Europe and in Algeria, but not one case occurred that year in Spain by reason of vigorous quarantine. Of this the speaker is personally cognizant, having there experienced a most villainous lazaretto—villainous from its want of comforts, but effectual from its restrictions. A year or two later the embargo was less strictly maintained, and cholera ravaged the Spanish

Peninsula. Those whose professional memory extends back to 1836, and those younger who have made the history of disease a study, will recall the vague notion which prevailed as to cholera. It was a something which always travelled from east to west. Ships sailing from a healthy port encountered it in midocean, and it struck and decimated those on board. Emigrants crossing the plains, bound for the just discovered El Dorado of the West, had their caravans mysteriously invaded by the fell destroyer; but time and observation dispelled these illusions, and it was found that the disease always followed the highways and byways of travel; that it was always carried by human beings, and although sometimes long in germinating, and greatly intensified by certain local surroundings, it could always be traced to individuals coming from some point of infection. If this be true of cholera, and it can be *fenced out*, is it not doubly probable that yellow-fever, with its more tangible elements of communicability, may be equally restrained?

Few quarantine stations are such as they should be. They should be made as pleasant and hygienic as location will allow. Usually required in the summer only, the buildings should be of wood, small, isolated, and with wide verandas, with all possible provision for ventilation, drainage, and necessary shade; and for these even suitable tents could often be advantageously substituted of materials which could be destroyed and renewed at but slight expense.

Quarantine, to be effectual, must have a very wide applicability; it will not do to limit it to vessels from foreign ports. It must extend to all conveyances for the transportation of passengers and merchandise—must have relations with municipal, state and national authority. As was here said two days since by the Health Officer of the Port of New York, at the meeting of the National Health Board: "Let the federal authority direct the sanitation, and the local powers take charge of all police regulations, and there will be no disturbance or disagreement."

A National Board of Health has been appointed, and it will doubtless be of great service, but it will probably not be vested with sufficient authority; its action will be chiefly advisory. There should be a National Health Bureau, with extraordinary powers for emergencies adequate to immediate action when required. It is at all times as necessary as any of the existing departments of State; and, if connected with the present Signal Service Bureau, as has long been the aim and desire of its chief, General and Dr. Albert J. Meyer, there is scarcely a limit to its usefulness.

If the question of expense is raised, let the answer be in the cost of the late epidemic. It has been computed that the actual money loss occasioned thereby amounted to the enormous sum of $200,-000,000. If we admit that the estimate is too large, and cut it in two, how small a proportion of this sum would suffice for all quarantine expenses, and for all contingent commercial deficits! The object of the preceding remarks must be apparent. It is to induce this body to use all its influence toward promoting the establishment of a National Health Bureau, not as an ephemeral health board to meet a sudden emergency, but as a permanent and essential part of a wise and provident government.

### HOW TYPHOID FEVER IS PROPAGATED.

The communicability of typhoid fever, through water supply tainted by the alvine evacuations of those affected by the disease, is now almost universally accepted as an established fact. It is a point of much interest—not as well known as it should be—that this was first brought to light thirty-five years ago, and published in the *American Journal of Medical Science* for July, 1845, by Austin Flint, M. D., then a resident of Buffalo. But, although distinctly made evident through his researches and investigations, it does not appear that he then recognized or suspected what is now very apparent.

A stranger from Massachusetts died at the tavern in North Boston, Erie Co., N. Y. He had been before unknown in that locality. He came on the 21st of September, having been ill for several days, and was unable to continue his journey. He died on the 19th of October. Between October 19th and December twenty-eight of the forty-three persons comprising the little community at North Boston were attacked with the fever, and in ten instances the disease proved fatal. Three families only in the settlement escaped. These did not obtain their drinking water from one common source; all the others did—from a spring on the grounds of the tavern (?). This being observed by the *people*, the belief was soon established that the fatal disease was not a fever, but that the spring had been poisoned by one of the exempt families who were at feud with the innkeeper. It was for this reason chiefly that Dr. Flint was sent for and employed to examine the nature and origin of the disease. From its character, and especially from a post mortem examination, he was enabled to pronounce it typhoid fever. The water from the spring was sent to competent chemists, who found it remarkably pure, and thus the theory of poison was disposed of; and yet with the light of the present day it can scarcely be doubted that it was contaminated with that deadly sept whose effects and whose mode of propagation we recognize, but cannot otherwise detect or define.

Dr. Flint's paper is entitled "Transportation and Diffusion of Typhoid Fever." He argues very closely and ably to demonstrate the communicability. He brings out the prominent feature of the supposed poisoned spring, and of those only being taken ill who drank from it. How nearly he escaped a grand discovery! But the facts which he then brought out and recorded, as now interpreted, are clearly the very first in which there is any mention of diffusion by contaminated water supply. Within the last ten years many instances confirming this view have been cited from various quarters, and notably the celebrated English dairy case, where the milk was the vehicle of the disorder, the cows having lain upon ground manured from infected vaults; portions of the excrement undoubtedly clung to their udders, and thus became directly mingled with the milk. Other explanations have been offered, but this is presumably the simplest and the correct one.

In the *Popular Science Monthly* for February, 1879, Ely Van De Warker, M. D., of Syracuse, N. Y., under the title "Typhoid Fever Poison," reports seventeen cases of the fever in an isolated suburb of the city, in which there were but fourteen houses. The first case was imported, thence through the overflowing of the privy in which all the excrement of the patient, had been thrown. A well became contaminated, all the persons who were taken ill used this well. It was the constant or occasional source of supply of seven of the fourteen families. No cases occurred in the households who did not drink from this well. Some cases were developed in every family who drew water from it. The families who escaped were exposed to every other influence but that of this particular well; their own water supply was the same, less the privy contamination. It is not unlikely that their own wells received some of the overflow from their own vaults, but as these were free from typhoid poison, no ill results ensued.

About eight years since the speaker became satisfied that a source of typhoid fever existed which was little dreamed of, and which at first thought would seem impossible. This source, as he then enunciated it to his home medical society (and not to his knowledge having been before suggested), is found in *ice*. If this idea is thoroughly investigated, it will not appear to be very problematical. In the first place, the poison is not destroyed or impaired by freezing (some one long ago remarked that ice often masks or conceals what it does not kill). Now, whence comes our ice supply? Often from shallow reservoirs in the midst or neighborhoods of large towns purposely made to receive surface drainage from all around, under the erroneous idea that no harm will ensue, as freezing is supposed to purify and render harmless what might othewise be objectionable. Great quantities of ice are taken from canals, from creeks, from stagnant ponds, and from streams that are either the natural or artificial recipients of surface drainage, of the outpourings of sewers, and of uncleanliness from various sources. The danger from ice taken from improper places is not only from that which is drank, but from its use in refrigerators and preservatories, where milk, butter, fruits, vegetables, and meats are subjected to its saturating influence as it vaporizes. Several instances have fallen under the speaker's observation where the disease, by the most careful investigation, could not be traced to any other source; and if we accept as a fact the statement positively made by Budd in the London *Lancet* in July, 1859, that it never originates, *de novo*, but proceeds from a special and specific poison, which is capable of diffusion to a great extent, and which preserves its noxious qualities for a long period, even if buried for many months, we cannot reject the hypothesis of ice infection; and it is hoped that it will be made the subject of very thorough and careful investigation. A statement is made by Liebermaister that it is by no means certain that recent immediate alvine dejections are more potential or active in extending the disease than those which have been exposed for some time upon refuse heaps, or have been long deposited in privies or buried deep beneath the surface.

In England the idea of the preventability of typhoid fever is now so thoroughly established, that an innkeeper, who had a guest ill with it, was notified that he would be held criminally responsible if any other case could be traced to the one under his roof. This, of course, implied, that the evacuations should be so thoroughly disinfected or destroyed that no harm could come from them—it could mean nothing else. It is claimed that the propagating property is destroyed by the application of boiling water to all the discharges, whether in vessels or on the bed or body clothing, and also indicates that other methods of disinfection should be employed. This is easily accomplished, and if the disease be recognized early, might be sufficient. But in many instances it would not be faithfully performed, and the morbid *ejecta* would be carlessly distributed in the usual receptacles. Can these be made innoxious?

It is claimed they can be, and not only rendered harmless, but useful, by a process now in operation in England, called precipitation. The following letter on this subject explains itself:

(COPY.)

COVENTRY, February 19th, 1879.

"DEAR SIR: In reply to your communication, I beg to inform you that the River Precipitation Association have been treating our sewage for nearly two years in a satisfactory manner. The principal is one of chemical precipitation by sulphate of alumina and other chemicals, and the effluent water or sewerage is passed over about nine acres of land as a simple final process of filtration. There are several villa residences adjoining the works and upon the banks of the river into which the effluent passes, but no complaint has ever been made by the occupants of any nuisance arising from the river, although the latter is a very small one—in fact, little more than a brook, the sewage in dry seasons being double its volume. I enclose a pamphlet," etc.

The pamphlet alluded to was read before the Congress of the Sanitary Institute of Great Britain in October, 1878, by Henry Robinson, F.R.G.S. The important portion is as follows: In the writer's experience in advising with reference to precipitation works and systems he has seen more and more clearly that sulphate of alumina, aided by milk of lime, proves the most efficient agent for purifying sewage. This he has now advanced upon, inasmuch as he has discovered that the efficiency of sulphate of alumina is greatly increased by the presence of some proto-sulphate of iron, which apparently produces results altogether out of proportion to its chemical equivalent, the proportion varying with the compensation of the sewage, but their combination having a marked sanitary and economic advantage. . . . The process previously employed gave highly satisfactory results, but the addition of the salt of iron has led to the production of an excellent effluent, and at less cost than previously. The employment of the combination has also resulted in the tanks and the sludge being deprived of the slight smell hitherto noticeable at times. The economic results attending this compound system of precipitation will be recognized when the writer states that "at the county sewage works the purification of the very foul sewage (containing between six and seven grains of chlorine per gallon, and between twelve and thirteen grains per million of albumenoid ammonia, with a large amount of dye from the manufacturers) is now accomplished for £1 1*s.* 1*d.* per million gallons. The sewage is equivalent to fifty gallons per head of the population per diem, the maximum flow being two and a quarter million gallons a day. The necessity for the adoption of such a standard of flow cannot be too strongly insisted upon, as the most fallacious estimates of sewage treatment by every system have been arrived at through ignorance or neglect of this essential consideration." The writer then goes on to explain how, by a process of pressure and filtration he converts the fluid sludge into a solid and portable form, which is readily purchased by farmers for fertilization, and without any insalubrious after-effects. All medical men are familiar with the deodorizing power of sulphate of iron; and if in the combination described it becomes a disinfectant, we have what is generally needed—a very cheap one.

It does not appear from the above report that it produces such a solid precipitation as to preclude its use in drains, privies, and sewers. One fact is very evident; that no sewer should be allowed to discharge into any body of water directly, but should empty into tanks or sluices, whence, after disinfection, it may pass into a pond or stream.

When a startling discovery is made or announced, its influence is apt to be exaggarated, and its application is often extended to subjects with which it has nothing to do. This is doubtless the case with reference to impure water supply. It is credited with too many evils; but that it will increase and intensify disorders springing from other causes and propagated by other channels cannot be doubted. Of this a very remarkable illustration has been afforded in an epidemic of diphtheria in Geneva, N. Y., during the past winter. The village is delightfully located on the banks of Seneca Lake. It stands high for the most part, on a bluff forming the western shore, and has always been considered healthy. Diphtheria invaded it during the winter months, and was characterized with unusual tenacity and fatality, subsiding at times and then recurring with great violence.

Investigation as to local causes was conducted by Prof. Breneman, of Cornell University, who found that while the water supply to some portions of the village was of the very best character, as that coming from the "White Spring," and which yielded only $\frac{2}{100}$ grains of chlorine to the gallon, the public pump on the corner of Main and Castle streets furnished 21 grains of chlorine to the gallon, exceeding the average of that furnished by the sewers of Boston and of some other cities. Other pumps and wells yielded 14, 12, 9, 8, 7 grains chlorine per gallon; and corresponding with the amount of chlorine was always a relationate quantity of albumenoid ammonia and of nitrates. Water from eighteen sources was examined, and thirteen were condemned.

The disease was more prevalent and much worse in the localities supplied with the bad water than in the others, but was not confined to them. A practical remark of much value, pertaining to the modification and propagation of disease as gathered from the condition of the water supply, is made by Prof. Breneman, to this effect: "It shows the extent of soil saturation and indicates the condition of the ground, the emanations and exhalations from which taint the air in which the families are obliged to live, and exposes them to a constant morbific agency."

Is not this a wise and admonitory deduction? and does it not explain the insalubrity of many localities of which a casual examination would not lead to the slightest suspicion.

### SANITARIA FOR TREATMENT OF PHTHISIS PULMONALIS.

So much interest was elicited last year by the remarks of the speaker's predecessor on "Climatic Treatment of Pulmonary Phthisis" that it is hoped a few words on the same subject may not be out of place. It is well known that in Europe mountain resorts for winter as well as summer are now on trial. Those most noted are at Goerbersdorf, Falkenstein, and Davos. A personal friend and patient who spent nearly two years in Colorado is now at the last mentioned resort for the second winter, and has sent so full a description of the mode of life and of its atmospheric surroundings—such as has not been published in any medical work or journal—that deductions have been drawn as to a probable cause of improvement not necessarily depending upon any special locality, and this is, *systematic and persevering out-of-door exercise in all kinds of weather.* To this there are undoubtedly adjuncts, which will be briefly considered. The village Davos am Platz, in Switzerland, is situated on a large plateau 5,000 feet above the sea level, and completely surrounded by mountains, which screen it from high and penetrating winds. Like that of almost all corresponding altitudes, the air is very dry; its humidity being less by two-thirds than that of Nice or Cannes. This gives it a property of elasticity, or, as it is sometimes, called, electric tension, which modifies in a remarkable manner the sensations of heat and cold. Persons often sit in the open air, even after sunset, with the mercury below freezing, without overcoats, without the slightest discomfort, and most of these invalids at that. The daylight at Davos is very short in winter, the sun overtopping the mountains about 10:30 A. M., and sinking behind their summits from 3:30 to 4 P. M.; but the sunshine, while it lasts, is very bright, and the heat of its direct rays is intense, rising to 100° or over, while at the same time the temperature in the shade will vary from 22° to 30°. The average temperature for Davos for January, 1878, was 16° most of the nights, and many days marking zero or below. To quote: "The sun will almost broil one's back, while icicles from his breath will form on his moustache and beard, and, changing front, his face will be actually sunburnt. From the constant sun-bronzing to which they are subjected, invalids soon lose all appearances of delicate health. The food generally is not inviting, nor even well-cooked, but an enormous appetite is established after a few days' sojourn; and the rule, to which there are but few exceptions, is that everybody gains more or less rapidly in flesh. The doctors drive every body out of doors—it is not too much cold, but too much heat that they dread.

"You may think there is plenty of time for correspondence, but this is not so—we are walking, sledging, coasting, skating, snow-balling, and eating. An excursion of ten miles on foot soon becomes a bagatelle to delicate women, who at home could not be induced to walk a fourth of that distance. In the evening there are hops, amateur theatricals, and various amusements—but all by medical direction—of short duration, which is even extended to the Sunday service, which is restricted to one session of one hour. Persons who have had hemorrhage cease to bleed, coughing and expectoration rapidly diminish- -in fact, there will be more coughing in an average country church than in one of our assemblies of invalids. Of course, all who come here do not improve; many are brought hither in a dying condition, but some of those apparently in that state, experience wonderful improvement, and pick up to a surprising degree; and, as little or no medicine is administered, the credit must be given to fresh air, locality, and exercise. Those who wilfully devote most of their time to cards, billiards, and saloons, where the air is made close by crowds and tobacco, do not generally improve much in health. The local physicians are very cheerful and enthusiastic. They talk a good deal about infiltration, consolidation, and cicarization of cavities, etc.; and as almost all the visitors are, or suppose themselves to be, consumptive, they catch these and other expressions, and speak so complacently of the changes their lungs are undergoing that we have to add a large stock of faith and hope to the natural curative properties of this region. Hotels and boarding-houses are becoming too numerous. A few years since there were only two. In fact, we are beginning to suffer some of the inconveniences that arise from too great an influx of people, and some of the medical men think that it may introduce elements of insalubrity as an almost unavoidable result of too much crowding."

The foregoing abstract of remarks made by an unprofessional but intelligent observer, and a self-watchful tubercular invalid, while it unfolds the daily life and usuages, and therefore here presented, coincides quite closely with the professional papers of T. Clifford Allbutt, of Leeds, published in the London *Lancet* within the past year. One of them commences with these words:

"For many years I have held that the majority of phthisical patients die of septicaemia, and that the arrest of this daily repoisoning is a primary object of treatment. . . . If there be an antiseptic climate, we may hope to counteract this secondary blood poisoning by sending our patients to live in it. . . . The antiseptic state of the air on the Alpine heights has been proved by Prof. Tyndall's experiments at Bel Alp; but that is insufficiently sheltered. In Davos alone, so far as we yet know, is found an air at once aseptic, bracing, and still. . . . There is given here, by implication at least, the impression, and by other writers the positive statement is made, that bacteria have much to do with the march and difusion of phthisis. It is suggested by the writer that these microscopic entities have altogether too much blame attached to them. They appear to be a component part of the tellurial atmosphere. They are found everywhere, but in varying proportion, and are quite as likely to be nature's air *scavengers* as the product and producers of morbific action."

They are credited with generating vibriones, micrococci, and microzymes, etc. Just now it may seem very heterodox to assert that, like other scavengers of larger size, they simply congregate where is found what it is their office to remove. In a thoughtful and suggestive paper published in the *American Medical Bi-Weekly*, entitled "A Few Words About Health Resorts," Dr. F. I. Bancroft, of Denver, Col., takes the strong ground that there is no place on the habitable earth that can bear the afflux of large and repeated numbers of tubercular patients without itself becoming a source and propagation of phthisis, no matter what its previous condition as to salubriety may have been, and no matter whether it be high or low, cold or warm, dry or moist. He quotes with approbation the following words from the *Medical and Surgical Reporter*:

"The time was when phthisis was practically unknown in Naples, in Madeira, in Malta, along the Rivera and the upper Nile. Consumptives flocked here in crowds, and now in all these districts the native population succumbs to the disease in quite as large proportion as elsewhere."

Dr. Bancroft asserts that tubercular phthisis is communicable by the breath and sputa, and by infection of apartments, and made more so by the insanitation of towns, hotels, boarding-houses caused by aggregation of diseased persons.

It is not news to any one here present, that this doctrine has long been held in Southern Europe. Dr. Bancroft claims that the advantage of one place over another consists in sparseness of population and in natural pureness of air, which is not easily contaminated. He recommends tent and cabin life, with gradual, and, so to speak, educated approach to the higher altitudes, and says in conclusion, "In Colorado, and I presume in all health resorts, out-door life is the most powerful factor for good. . . ." After all, it is going back to the example set by the first Dr. Parish, of Philadelphia, who, on coughing and spitting blood and emaciating when a young practitioner, discarded his chaise and made all his visits on horseback, and thereby regained and preserved his health, living to a good old age. Proper health resorts, with judicious regulations, will do something, but they are necessarily only for the few. What shall be done for the great multitude who cannot leave home? What but less of medication and more of hygiene—hygiene in its largest sense—embracing occupation, habitation, good nutrition, marriage relation, and, with all the rest, and above all in its importance, systematic and practical in-door and out-of-door judicious exercise.

